# Narrative text comprehension: how good and poor reading comprehenders use general working memory and specific semantic resources

**DOI:** 10.1007/s10339-026-01365-1

**Published:** 2026-06-15

**Authors:** Caterina Artuso, Carmen Belacchi

**Affiliations:** 1 University of Genova, Genova, Italy; 2 University of Urbino Carlo Bo, Urbino, Italy

**Keywords:** Reading comprehension, Narrative text, Individual differences in comprehension, Semantic organization, Working memory

## Abstract

In this study, we investigated the contribution of verbal working memory (WM) and semantic organization resources in poor and good reading comprehenders (age range: 7.5–11 years). From an initial sample of 151 children, 20 poor and 18 good reading comprehenders were selected and administered a battery of WM tasks -ranging from low to high attentional demands (forward span, backward span, selective word span, and dual task word span)- as well as a semantic WM task involving the manipulation of taxonomic and thematic semantic organization. The findings support the role of working memory (WM) attentional resources, particularly those implicated in the backward span task, in differentiating between skilled and less-skilled reading comprehenders. In addition, a specific contribution of taxonomic and thematic semantic resources emerged. Notably, we observed a correlation between good reading comprehension and stronger taxonomic associations, whereas this relationship was absent in poor comprehenders. Taken together, these findings suggest that specific semantic memory resources contribute to reading comprehension over and above general working memory capacities.

## Introduction

In the current study, we investigated the role of verbal working memory resources -specifically general attentional working memory (WM) and semantic working memory (SWM)- in primary school children who are skilled versus less-skilled reading comprehenders. Our aim was to disentangle the contribution of distinct WM components by employing a battery of tasks varying in attentional control demands, while also examining semantic memory through the distinction between taxonomic and thematic organization.

Although the role of WM in reading comprehension is well established, the contribution of semantic working memory (and, more specifically, the type of semantic organization involved) remains underexplored. Building on evidence that taxonomic and thematic relations differentially affect WM performance across populations (Belacchi et al. 2011, 2022; Artuso et al. 2020a, b; Artuso et al. 2022), the present study examined their distinct contributions to reading comprehension. We hypothesized that these components would differentially support the performance of skilled and less-skilled reading comprehenders, in line with findings from adult populations (Artuso and Belacchi 2021).

Overall, this study provides a more fine-grained account of the cognitive mechanisms underlying reading comprehension by integrating attentional and semantic components of WM. These findings have implications for theoretical models of comprehension and may inform the development of targeted interventions for children with reading comprehension difficulties.

### Working memory and semantics in reading development

Reading comprehension emerges from a complex integration of multiple skills, including decoding (Torgesen 2000), vocabulary (Verhoeven & van Leeuwe, 2008), syntactic (Oakhill and Cain 2012), and semantic knowledge (Nation et al. 1999). In addition, it relies on higher-level control processes (Cain 2006; Kintsch 1988), among which working memory (WM) is one of the most robust predictors in both adults (Daneman and Carpenter 1980) and children (Cain et al. 2004). Complex span tasks (requiring both storage and active manipulation of information) have been shown to predict reading comprehension more strongly than simple storage measures.

Beyond WM, semantic resources play a crucial role in supporting comprehension. Semantic processing skills have been shown to contribute to reading comprehension in childhood and to predict later outcomes (Nation and Snowling 2004), with evidence suggesting that depth of vocabulary knowledge is a stronger predictor than breadth and may mediate the relationship between WM and reading comprehension (Artuso and Palladino 2022). Similarly, retrieval-based measures such as semantic fluency account for longitudinal variance in reading comprehension development (Nouwens et al. 2018), and semantic WM measures have been found to be more predictive than phonological storage capacities (Nouwens et al. 2017).

Semantic memory is commonly described as comprising two main organizational systems: taxonomic and thematic (Mandler et al. 1987). Taxonomic organization groups items based on shared intrinsic features within hierarchical, decontextualized structures (e.g., animal–lion), whereas thematic organization links items through contextual or experiential relationships (e.g., knife–meat). Developmental evidence suggests that thematic associations support memory performance in early childhood, while taxonomic organization becomes more prominent from around age 7 onward and across the lifespan (Belacchi et al. 2022). This shift likely reflects increasing cognitive efficiency and abstraction abilities, as taxonomic structures facilitate the organization and integration of semantic information.

Importantly, taxonomic organization may be particularly relevant for reading comprehension, as it supports the formation of coherent mental representations of text by enabling the grouping of semantically related concepts, promoting abstraction, and facilitating inferential processes across sentences and discourse units. In contrast, thematic associations may be more closely tied to context-bound or experience-based processing. Moreover, the use of taxonomic and thematic relations is influenced by task demands: explicit tasks tend to elicit thematic associations, which are more accessible, whereas implicit tasks reveal earlier sensitivity to taxonomic structures (Hashimoto et al. 2007). Developmentally, the ability to use taxonomic relations increases with age but may decline in older adulthood (Belacchi and Artuso 2018).

Despite the established role of semantic memory in reading comprehension, relatively few studies have explicitly distinguished between taxonomic and thematic associations in this domain. Existing evidence suggests that semantic associations support reading comprehension by activating relevant background knowledge (Myers & O’Brien, 1998), and that different types of semantic knowledge may be differentially recruited depending on age and task demands (Artuso and Belacchi 2021). However, it remains unclear whether and how these two types of semantic organization differentially contribute to reading comprehension in children.

### Working memory and semantics in good and poor reading comprehenders

Poor comprehenders are readers who struggle with reading comprehension but usually do not show phonological, word reading impairments (e.g., Sleeman et al. 2024). Comprehension abilities in poor and good comprehenders have been studied with reference both to WM resources and semantic resources. With reference to WM resources, Palladino et al. (2001) found a specific relation between WM updating and reading comprehension in individuals aged 11–21 years. Updating is a mechanism used in maintaining relevant information and removing information that is no longer relevant. The authors (2001) showed that poor reading comprehenders’ WM recall was lower than good comprehenders; in addition, poor comprehenders produced a greater number of intrusion errors, an index of low WM updating abilities. In the same vein, Borella et al. (2010) showed that poor reading comprehenders aged 10 to 11 years (relative to good ones) were impaired both in WM tasks and in inhibitory tasks, suggesting a more nuanced view of inhibition-related impariment.

With reference to semantic resources, Nation and Snowling (1998) compared poor and good reading comprehenders on a set of phonological and semantic processing skills (mean age 9.3 years). The two groups performed at a similar level on phonological tasks; however, the poor comprehenders performed less well on tasks targeting vocabulary knowledge and semantic processing skills, including a semantic retrieval task. The authors proposed that reading comprehension difficulties may be explained by the delay or inability to access word meanings.

In addition, Nation and Snowling (1999) reported differences in sensitivity to semantic relations between poor and good comprehenders (aged 6 to 10 years). The authors employed a semantic priming task targeting taxonomic relations (e.g., *cat–dog*) and thematic relations (e.g., *floor–broom*). Good comprehenders exhibited priming effects for both taxonomic and thematic targets, whereas poor comprehenders showed reliable priming primarily for thematic relations. Crucially, taxonomic priming differentiated the two groups, as it emerged independently of associative strength only in good comprehenders. These findings suggest that poor reading comprehenders rely more on thematic associations, whereas good comprehenders more efficiently process taxonomic relationships.

Carretti et al. (2013), in a sample of children aged 8 to 10 years, showed that poor comprehenders’ writing performance was generally worse than that of good comprehenders in narrative text production, but comparable in descriptive text production. The authors argued that reading comprehenders’ difficulties are related to the characteristics of the narrative text where coherence and causality are important elements (see also Myers & O’Brien, 1998). In sum, different studies highlighted the difficulties that poor comprehenders show with both the narrative text comprehension and production, as well as their overall WM weaknesses.

From a theoretical perspective, these findings can be framed within models of text comprehension that emphasize the construction of a coherent situation model (e.g., Kintsch, 1998). Narrative texts, in particular, place high demands on processes such as the integration of information across the text, the generation of bridging and elaborative inferences, and the continuous updating of the mental representation in WM. Crucially, these processes overlap with the core weaknesses observed in poor comprehenders, including limitations in WM updating, inhibitory control, and the organization and access of semantic knowledge. As a result, poor reading comprehenders are less efficient in constructing and maintaining coherent situation models, especially when causal relations and global coherence must be inferred rather than explicitly stated. This theoretical alignment helps to explain their specific difficulties with narrative texts and highlights the central role of integrative and inferential processes in successful comprehension.

### The present study: rationale and aims

We aimed to explore the role of different memory resources in reading comprehension of narrative texts in good and poor comprehenders. We distinguished and assessed WM (via a verbal battery of more nuanced tasks to tap a continuum in the allocation of attentional cognitive resources) and semantic resources (via a semantic WM task). With respect to general WM, we started with low-control tasks (forward span), low-to-medium control (backward span), medium control (selective word span), and high control (dual-task), following the Cornoldi and Vecchi (2003) model.

Cornoldi and Vecchi (2003) proposed the distinction between WM tasks according to their level of attentional control. The authors argued it is possible to distinguish WM tasks by reference to the degree of attentional control, and stated that, within the WM tasks themselves, the degree of controlled activity can vary along a continuum. Therefore, each memory task requires a certain level of control activities, the degree of involvement depending on the task demands. The authors proposed a distinction between passive processes (e.g., simple recall of previously acquired information), and various degrees of active processing (i.e., manipulation of information to produce an output different from the original inputs). Less active tasks, or passive memory tasks, such as simple span tasks (i.e. forward digit span), require a very low degree of controlled processes, as individuals have only to reproduce the item just presented; therefore, here, the mere rehearsal of items is usually sufficient. A task such as the backward word span comes next, as it is assumed to require slightly more control; here, the individuals must perform a simple operation on the material (i.e. reverse the word order). Tasks at a high level of active processing are those requiring active processing and temporary maintenance of information, such as the WM dual-tasks, and the updating tasks. Confirmation to this model comes from studies with typically developing children (Belacchi et al. 2010) and clinical populations (e.g., individuals with Down syndrome, see Lanfranchi et al. 2004; Carretti et al. 2010).

To evaluate semantic resources, we used a dual task modeled after the complex span tasks devised by Daneman and Carpenter (1980) where the role of taxonomic and thematic semantic memory was considered. Indeed, though the semantic contribution to comprehension is quite clear (e.g. Nation and Snowling 1998; 1999; Nouwens et al. 2017). Previous studies have largely overlooked the distinction between taxonomic and thematic organization, with the notable exception of Nation and Snowling (1999), who used a semantic priming task to assess sensitivity to different types of semantic relations. Most of the studies considered semantic retrieval (measured via fluency tasks, e.g., Nouwens et al. 2017), semantic storage (e.g., Nouwens et al. 2017) and semantic vocabulary depth (Artuso and Palladino 2022).

To our knowledge, there are no studies that specifically investigated the association between narrative text comprehension and the semantic organization (i.e., taxonomic and/or thematic) with an explicit WM task in developmental age. We were interested in study the role of different memory resources during narrative text comprehension. Specifically, our purpose was to point out the role of memory resources (general and semantic WM) in good and poor comprehenders.

Overall, we predicted better performance in good comprehenders compared to poor comprehenders across both types of tasks, in line with previous research (e.g., Carretti et al. 2013; Nation and Snowling 1999). Regarding the role of general attentional resources, we expected stronger associations with the WM tasks requiring higher attentional control. As for semantic resources, we hypothesized a greater involvement of taxonomic representations, which enhance the activation of abstract categorical information and, in turn, promote inference-making abilities -particularly relevant to reading comprehension- as also found by Nation and Snowling (1999).

To this end, our findings would support and extend findings from Nation and Snowling (1999) using a different, explicit task, and in the same age range. In addition, in line with previous literature, children with learning disorders have been shown to exhibit weaker executive functioning -particularly in attentional control (e.g., Capodieci et al. 2023)- as well as specific difficulties in managing linguistic information, especially abstract taxonomic representations (Artuso et al. 2020a, b; Hashimoto et al. 2007). Based on this evidence, we expected that poor comprehenders would rely less on general attentional resources and would favor thematic over taxonomic semantic categories.

## Methods

### Participants

A total of 151 school children aged 7.5 to 11 took part in the study. From that original sample, 20 poor comprehenders (11 female) and 18 good comprehenders (8 female) were identified. The groups were matched on fluid intelligence using Raven’s Progressive Matrices (Coloured Progressive Matrices- CPM, see below). The groups were created by selecting children who in narrative text comprehension fell below the 25th percentile (poor comprehenders), and those who fell above the 75th percentile (good comprehenders) (see e.g., Borella et al. 2010; see also Sleeman et al. 2024 for the impact of different cutoffs on the identification of poor/good comprehenders).

Children were recruited from several public schools in central Italy, located in areas with mixed socio-economic backgrounds. All participants were native Italian speakers. Children with a certified diagnosis of a learning disability or other neurodevelopmental disorders were excluded from the sample.

Participants were tested at school by an appropriately trained Master’s student. Written informed parental consent, as well as oral informed child assent, was obtained and collected before participation, according to the ethical norms at our universities. The study was conducted in agreement with the Ethical Standards of the Declaration of Helsinki and those recommended by the Italian Psychological Association.

### Materials

#### Coloured progressive matrices - CPM

In this standardized test, used as a measure of fluid intelligence (Raven 1992; for Italian norms see Belacchi et al. 2008), participants are individually presented with 36 visual patterns (with one piece missing from the bottom right corner), grouped into three sets of 12 items each. For each visual pattern, participants are presented with various alternative pieces that could complete the table and must choose the correct one. They are instructed to answer each question before moving to the next table. No time limits are given. Participants must attempt all 36 items of the task, the final score being the sum of correct answers across the three series.

#### WM attentional resources

We used a battery consisting of four verbal WM tests, developed by Lanfranchi and colleagues (2004). The original battery is composed of verbal and visuospatial tasks. Here, we used the verbal section only. The tasks differed in the amount of requested controlled attention: from the simplest (e.g., forward span, backward span) to the more complex tasks (selective word span, and dual task). In addition, in each task an increasing memory load has been manipulated: in the forward span 2–7; in the backward span 2–6; in the selective word span 1–5, and in the dual task 2–5 (see full description below).

In the *forward span* (low control), lists of 2–7 words were presented to the participant who had to repeat the list immediately and in the order of presentation. In the *backward span* (low-to-medium control), lists of 2–6 words were presented; participants were then asked to repeat each list in reverse order immediately after presentation. One point was awarded if the sequence of words was repeated in the same order (or reverse) of presentation, and 0 if the words were incorrect, incomplete, or presented in different order. The final score was obtained from the sum of the single scores obtained for each series and ranged from a minimum of 0 to a maximum of 7 for the forward span and 6 for the backward span.

In the *selective word span* (medium control), for each level of memory span (from 1 to 5), two lists were presented to the child, who had to recall the first word of each list after the presentation of the entire series. In the first span level, the child was presented with two 1-word lists; in the second span level, with two 2-word lists; in the third span level, with two 3-word lists, in the fourth span level, with two 4-word lists, and finally in the fifth span level, with two 5-word lists. For example, the second trial was as follows: *cat-granny*, followed by *ball-cow*. Here the child had to remember the words *cat* and *ball*. One point was given for each series correctly recalled for a maximum score of 10.

In the *dual task word span* (high control), lists of 2–5 words (one of which was an animal name) were shown to the participant who was asked to remember the last word on the list and to tap on the table when an animal noun was presented (secondary task). For each level of memory load (from 2 to 5), two trials composed of two lists were presented to the child. Thus, at level 2, in the first trial, the participant was presented with two 2-word lists, in the second trial, with two 4-word lists, at level 3, with three 2-word lists and then three 4-word lists, and so on. Participants were informed they were wrong if they remembered the word correctly but forgot to tap. The score ranged from 0 to 8.

To facilitate understanding of the instructions, practice trials were provided for each task. In all tasks, words were presented verbally at a rate of one per second. Tasks progressed from shorter to longer trials, with two trials per level. To minimize frustration, testing was discontinued if participants failed both trials at the same length; remaining items were scored as incorrect. All tasks showed good reliability (0.70–0.90; see also Belacchi et al. 2010). Performance was the number of correctly recalled words across tasks.

#### Semantic organization resources

To specifically target semantic taxonomic and thematic resources, we used the Semantic WM task (SWM) devised by Belacchi and Palladino (2017). The materials consisted of word trials (*N* = 60), each containing four words; out of the four words, one was always a digit (from 1 to 9; never in the last position). In each trial, words were combined according to three different types of semantic association, in the same proportion (i.e., 20 trials for each association): (i) arbitrary association, with unrelated words (e, g., coffee, car, book); (ii) taxonomic association, with words belonging to the same class, specified by a taxonomic superordinate term, always presented in first position (e.g., color, yellow, blue, where color is the superordinate term); (iii) thematic association, with words contextually related to each other (e.g., water, salt, sea).

The 60 trials were administered in random fixed order and arranged in sets of increasing spans (i.e., spans 2 to 6). Words were controlled for lexical frequency, word length, and imageability, and were all positioned in the medium-to-high frequency range within the Italian primary school lexicon (Marconi et al. 1994). The words were controlled for frequency of use in the elementary lexicon, all falling within the medium-to-high frequency range (according to the norms provided by Marconi et al. 1994). In addition, we ran a control analysis to check word length. We compared the number of syllables across the three types of associations. Mean syllable counts were 2.3 for arbitrary words, 2.4 for thematic words, and 2.6 for taxonomic words. Paired-sample t-tests revealed significant differences between arbitrary and taxonomic stimuli (*p* < .001) and between taxonomic and thematic stimuli (*p* < .05). Despite being shorter, arbitrary words are more difficult to recall, whereas recall for taxonomically related words improves with age, despite their greater length (see Belacchi et al. 2011, 2022).

The task was structured as a dual task. Participants listened to each trial of four words spoken aloud by the experimenter, delivered with approximately 1 s interval after each word within the trial. They were required to (i) tap on the table whenever they heard a digit (secondary task), and (ii) recall the last word of several trials after hearing several trials of increasing spans 2–6 (primary task). A practice trial was presented at the beginning of the task to control for task instruction understanding and to familiarize participants with task procedures.

Accuracy was measured. Most participants performed the secondary task correctly (between 95% and 99% of correct responses). However, the secondary task (i.s., tapping when encountering a digit) was not specifically analyzed as the aim to have a digit detection task was only to overload WM serving as a distractor, and enhancing task complexity. See also Belacchi and colleagues (2022) for raising this issue.

Each answer received a score of 1, if correct, or 0 if incorrect. The SWM task has shown good reliability and concurrent validity: Cronbach alpha= 0.88, and a correlation of *r* = .40 with the forward span, of *r* = .44 with the backward span, and *r* = .56 with the selective word span (all *ps* < 0.001) (Belacchi and Palladino 2017). In the following example of a span 2 trial the participants must recall *punta* (‘tip’) and *tulipano* (‘tulip’) and tap on the table when they hear *uno ‘*(‘one’*)* or *sette* (‘seven*’).*

trial 1: *uno-madre-posto-punta* (arbitrary) ‘one-mother-place-*tip*’.

trial 2: *fiore-margherita-sette-tulipano* (taxonomic) ‘flower-daisy-seven-*tulip*’.

#### Narrative text comprehension

Reading comprehension was assessed by administering a single narrative text taken from the Italian standardized reading battery for children devised by Cornoldi et al. (2017). Each narrative text had about 170–180 words. The content of each text differed by class level; therefore, a different test was administered to the children in different grades. The Crombach alpha was calculated for the current sample, showing high reliability (alpha = 0.94).

A narrative text usually describes a sequence of events that are related to each other temporally and causally (Lichtenstein and Brewer 1980) and centers around one or more protagonists who engage in series of actions, to achieve a goal. Each participant was instructed to silently read the text (at her/his own pace). After reading the text, the participants were asked to answer text comprehension questions on a response sheet. According to the standard procedure, participants could review the text during the response phase.

Multiple choice questions followed the text reading: each question had four choices, with only one of them correct. The texts were followed by different questions depending on the class attended: ten questions for first and second grade, twelve for third grade, and fourteen questions for fourth and fifth grade. The questions engaged the participant with the literal or the inferential processing of text necessary to its understanding. The total number of correct answers was collected and represented as an index of reading comprehension ability. Accuracy was collected, that is the number of correct responses. Given the differences in number of questions (i.e., ten, twelve or fourteen), mean proportional scores were used.

#### Procedure

Text comprehension was administered collectively in each classroom at the end of the school year, when all children (including first and second graders) have acquired the basic skills required in reading simple texts. Afterward, participants were tested individually in two sessions: during the first session (lasting about 30/40 minutes) they were administered the CPM battery and the Semantic WM task. One week later, during the second session, lasting about 30 min, children were individually administered the WM battery: the order of task presentation was fixed to minimize any error due to participant-by-order interaction.

## Results

The two groups of poor (*N* = 20) and good (*N* = 18) comprehenders were matched for school grade (*t*(36) = 0.92, *p* = .37) and fluid intelligence (*t*(36) = 1.34, *p* = .19). Indeed, the mean average score for the Raven matrices was 28.28 (*SD* 3.74) in poor comprehenders and 30.11 (*SD* 4.97) in good ones. The groups differed only in their reading comprehension scores, *t*(47) = 12.91, *p* < .00 (poor comprehenders *M* = 0.58, *SD* = 0.07; good comprehenders *M* = 0.96, *SD* = 0.05).

A preliminary bivariate correlation on the whole sample (corrected by age) was run between variables to explore the relationship between the memory measures as well as their association with reading comprehension. Next, analyses comparing general and specific WM reosurces in good vs. poor comprehenders were conducted.

### The relationship between WM and comprehension: correlational analyses

Pearson moment inter-correlations were run controlling for class/age. As shown in Table 1, almost all measures of WM tasks are highly related with each other (all *ps* < 0.001). In particular, the comprehension task was associated with both WM and SWM measures; however, the association was negligible for the dual-task WM condition (*r* = .01) and weak for the arbitrary association SWM condition (*r* = .13). In contrast to the literature (see e.g., Daneman and Carpenter 1980), no correlation between the reading test and the dual task was observed (see Table 1, *r* = .04). We believe this could be related to the text comprehension task used that, in the Italian research context, represents a general assessment measure (thus, too easy) and not an advanced text to thoroughly study reading development processes. Nevertheless, we have decided to administer this text comprehension task that was adequate to the current exploratory study, lacking a direct comparison in the literature.

**Table 1:** Pearson-moment correlations among different WM measures and text comprehension controlling for class/age

1. Forward span	2.	3.	4.	5.	6.	7.	8.
	0.20**	0.47***	0.21**	0.26**	0.24***	0.23**	0.18**
2. Backward span		0.24**	0.06	0.33***	0.25**	0.18**	0.23**
3. Selective word span			0.39***	0.36***	0.46***	0.26**	0.22**
4. Dual task word span				0.24**	0.23**	0.18**	0.04
5. Semantic taxonomic WM					0.63***	0.59***	0.22**
6. Semantic thematic WM						0.60***	0.18*
7. Semantic arbitrary WM							0.13
8. Narrative text comprehension							**-**

All significance tests are two-tailed. ***p < .001, ** *p* < .01, * *p* < .05

### General WM resources and semantic WM: comparison between poor and good reading comprehenders

Two analyses were run to compare good and poor comprehenders in the two memory systems under investigation: a univariate analysis on the mean scores of the general WM measures; then, a repeated measures anova on the mean scores of the WM semantic task.

Univariate ANOVA analyses were conducted on the mean scores of the forward span, the backward span, the selective word span and the dual task word span (as dependent variables), and group (poor/good comprehenders) as independent variable. The only measure that differed between groups was the backward span, *F* (1, 36) = 11.88, *p = .*001, with a better performance for the good comprehenders, compared to the poor ones. All the other three measures did not differ between groups, *F* < 1. See Table 2 for full measures. Effect sizes (Cohen *d*) are reported.

**Table 2:** Descriptive statistics (means and standard deviations in parentheses), p values and effect sizes for WM measures in poor and good comprehenders

Measure	Minimum/ Max score	Poor comprehenders	Good comprehenders	*p* value	Effect size
Forward span	0–7	6.15(1.03)	6.60(1.33)	0.158	0.37
Backward span	0–6	3.10(1.33)	4.50(1.15)	0.001	1.12
Selective word span	0–10	6.70(1.60)	7.60(1.64)	0.121	0.55
Dual task word span	0–8	6.65(0.93)	6.67(2.01)	0.974	0.01

For the WM semantic task, scores were considered as dependent variables, and group as independent variable. Therefore, an analysis of variance with the semantic association as within participants factor (arbitrary, thematic, taxonomic), and the group of comprehenders as between participant factor (poor, good) was run. The main effect of group was significant, *F* (1, 36) = 5.44, partial η² = 0.13, *p* = .025; on average, good comprehenders (*M* = 12.24, *SD* = 0.50) scored better than poor ones (*M* = 10.55, *SD* = 0.51). The main effect of semantic association was significant too, *F* (2, 72) = 10.23, partial η² = 0.22, *p* < .001. On average, taxonomic associations (*M* = 12.36, *SD* = 0.31) are better recalled than thematic (*M* = 11.45, *SD* = 0.43) (*p* = .025), and arbitrary associations (*M* = 10.38, *SD* = 0.55) (*p* = .001). Furthermore, thematic association are better recalled than arbitrary associations, *p* = .008.

**Fig. 1:**
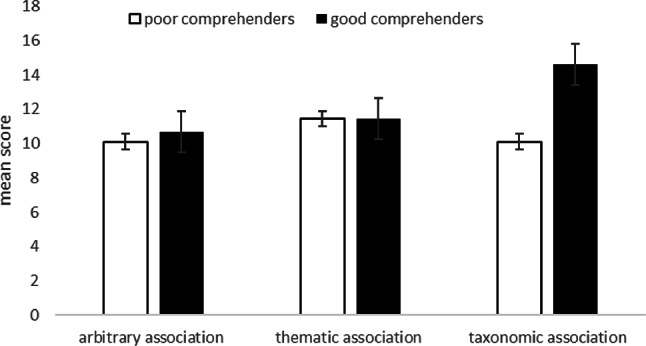
Semantic association scores by group interaction. Bars represent SEM (Standard Error of the Mean)

In addition, the interaction between group and semantic association reached significance as well, *F* (2, 72) = 15.88, partial η² = 0.31, *p* < .001. To further analyze the interaction, and related to our aim, we ran independent sample t-tests showing that the thematic association did not differ between groups, *t*(36) = 0.007, *p* = .99, CIs (-1.72- 1.73), *d* = 0.003, as well as the arbitrary association, *t*(36) = 0.51, *p* = .62, CIs (-2.81–1.69), *d* = 0.17. Only the taxonomic association produced better recall in the good reading comprehender group than in poor comprehenders, *t*(36) = 7.31, *p* < .001, CIs (-5.76, − 3.26), *d* = 2.40. See Fig. 1.

## Discussion

The main aim of the present study was to investigate the availability of working memory (WM) and semantic resources in poor and good comprehenders (see also Cain et al. 2004; Daneman and Carpenter 1980). The two groups were matched within each grade on fluid intelligence, ensuring that any observed differences were related to the specific WM resources examined, rather than to general cognitive abilities, which were comparable between groups. In line with previous findings (Nation and Snowling 1999; Palladino et al. 2001), we anticipated better performance in good comprehenders than in poor ones across both tasks. More specifically, we hypothesized that taxonomic semantic resources would differentiate the two groups. Overall, we confirmed the established role of general WM resources in reading comprehension and, more originally, highlighted the contribution of semantic resources in distinguishing between good and poor comprehenders.

We focused on taxonomic and thematic semantic memory, two components that are often overlooked in assessments of the semantic resources involved in comprehension (but see Nation and Snowling 1999). While the overall contribution of semantic knowledge to reading comprehension is well established (e.g., Artuso and Palladino 2022; Artuso and Belacchi 2021; Nation and Snowling 1998, 1999; Nouwens et al. 2017), in the present study we explicitly distinguished between thematic and taxonomic components using a novel semantic WM dual-task (Belacchi and Palladino 2017), modeled after traditional WM dual-tasks (see Daneman and Carpenter 1980).

A more detailed discussion of the assumption regarding the “fundamental cognitive component involved in text comprehension” appears warranted considering the present findings. Although good comprehenders outperformed poor comprehenders across all WM tasks, only the backward span task clearly differentiated between the two groups (see Table 2). This pattern is consistent with previous research (Goff et al. 2005; Daneman and Merikle 1996), suggesting a role for WM in reading comprehension. The backward span, commonly used to assess WM, is the only task among those administered that appears to involve a basic form of updating (namely, maintaining relevant information while discarding no-longer-relevant input) while also requiring the reordering of items. This combination of updating and manipulation may represent a key cognitive mechanism underlying text comprehension, as it supports the ongoing integration and restructuring of information during reading, consistent with prior findings (e.g., Palladino et al. 2001).

We did not find the expected contribution of high resource tasks though the literature shows the involvement of those tasks in reading differences (see work by Daneman and Carpenter 1980). We believe this finding could be related to the reading comprehension narrative test we used, which was a single text (see below, among limitations) and likely too easy to detect subtle differences.

In line with our expectations, we observed differences between good and poor reading comprehenders in the use of taxonomic memory resources: good comprehenders showed greater sensitivity to abstract categorical representations and derived greater benefit from them. These findings support the view that taxonomic organization -by enhancing the activation of abstract categorical information and supporting broader inferential processes- plays a key role in reading comprehension in primary school children.

As reported in Table 2, the backward span (as a measure of general WM resources) and the taxonomic semantic WM show the greatest significance values and effects sizes. However, it is worth noting that also the forward span and the selective word span show a medium effect size. Therefore, we believe that the absence of a clear significance (of the *p* values), might be related to the small sample size. Further evidence will be necessary to support our findings.

Our result fits with Nation and Snowling’s study (1999) who demonstrated greater sensitivity to taxonomies in good reading comprehenders via the administration of an implicit priming memory task. Here, in addition, we have reciprocally demonstrated that poor comprehenders show a difficulty in the use of taxonomies also in a semantic WM recall task, showing a less abstract semantic organization. Also, the present finding raises several issues concerning individual differences in the development of semantic memory. Compared to good comprehenders, the poor comprehenders appear to have less available abstract semantic categories based on ontological and logical knowledge; this suggests that their reduced reading comprehension could also be due to lower availibility of specific basic text comprehension requirements (such as inference making skills, and understanding sequences of events that are temporally and causally related to each other; e.g., Lichtenstein and Brewer 1980). The findings are also in line with the scarce use of taxonomic categories in children with developmental dyslexia (Artuso et al. 2020a), where WM recall was not boosted by taxonomy use, contrary to typically developing children who took advantage of the use of taxonomic categories. Additionally, further in-depth analyses of the relationship between reading comprehension and semantic knowledge showed that taxonomic knowledge supported comprehension in typically developing children, whereas children with developmental dyslexia relied more on thematic knowledge (Artuso et al. 2026). These findings highlight distinct cognitive profiles in developmental dyslexia and underscore the role of semantic organization in reading comprehension beyond decoding difficulties.

Therefore, we believe that this finding, though explorative, is original and relevant as it demonstrates the role of different semantic organization resources (taxonomic, thematic) on reading comprehension, beyond WM general attentional resources, as previously shown (e.g., Cain 2006; Cain et al. 2004). Indeed, previous work has typically limited the investigation of reading comprehension to general vocabulary breadth (e.g., Chrysochoou et al. 2011; Gathercole et al. 1992), often neglecting the potential contribution of more specific semantic resources—such as taxonomic and thematic knowledge—which deserve further attention. Except for a recent study by Artuso et al. (2025) demonstrating a link between definitional skills (conceptualized as taxonomically based verbal vocabulary knowledge) and listening comprehension in children from preschool to school age, this aspect remains largely unexplored.

Some limitations should be acknowledged. First, the use of a single text (likely too easy) may represent a shortcoming. However, in the present study, given the wide age range of participants, we chose to administer a text standardized by school grade. In future research, it may be useful to develop ad hoc texts as well. Additionally, the limited number of comprehension questions did not allow for a clear distinction between literal-descriptive and inferential-interpretative questions (e.g., Chrysochoou et al. 2011). In future studies, distinguishing between question types would be highly valuable to more thoroughly investigate the role of taxonomic and thematic organization in inference-making processes (e.g., local vs. global comprehension). We did not compare age groups due to the relatively small sample size; however, this remains an important avenue for future research. Increasing the sample size would allow for the inclusion of distinct and comparable age groups, which is particularly relevant given that taxonomic strategies in working memory are typically used from around age seven onward (Belacchi et al. 2022). This would, in turn, enable a comparison of the effects of different types of semantic organization on text comprehension before and after age seven, in both good and poor readers.

In sum, the relevance and originality of this contribution lie in having analyzed, for the first time in primary school children, as previously done with adults and older individuals (Artuso and Belacchi 2021), the contributions of different types of taxonomic and thematic semantic associations to text comprehension. This was achieved through a specific, explicit working memory task, thereby expanding upon previous findings obtained using an implicit semantic priming task (Nation and Snowling 1999).

However, the SWM task we used, designed to assess semantic WM, was not merely a measure of semantic knowledge. It would be of interest to include a more independent measure of semantic knowledge, unrelated to WM performance, to assess semantics more directly (e.g., through vocabulary access, categorization tasks, or other measures such as vocabulary breadth and depth). In addition, incorporating further reading-related measures, such as decoding skills or morpho-syntactic competence, could offer a more comprehensive understanding. The relationship identified between limited taxonomic organization and comprehension abilities likely operates in a bidirectional and circular manner. Only a longitudinal research design could clarify the potential causal relationship between the two and better specify the role of different semantic representations involved in reading—a crucial skill for learning and academic success. Our findings may help inform the design of targeted intervention programs aimed at fostering the development of semantic skills in poor comprehenders, who tend to use fewer strategies than their good comprehending peers (Kletzien 1991).
